# Molsidomine Pretreatment Modulates Renal Redox and Endothelial Activation Markers in Experimental Polymicrobial Sepsis

**DOI:** 10.3390/ijms27209088

**Published:** 2026-10-14

**Authors:** Adem Kose, Zeynep Busra Keser, Mehmet Gul, Seyma Yasar, Onural Ozhan, Zeynep Ulutas, Feyzi Dogru, Ezgi Kurucay, Ayse Betul Levent, Mehmet Ertugrul Balkar, Hakan Parlakpinar

**Affiliations:** 1 Department of Infectious Diseases and Clinical Microbiology, Faculty of Medicine, Inonu University, 44280 Malatya, Türkiye; zakpolat0944@gmail.com; 2 Department of Histology and Embryology, Faculty of Medicine, Inonu University, 44280 Malatya, Türkiye; mehmet.gul@inonu.edu.tr (M.G.); ezgikaraaslann@gmail.com (E.K.); betullevent@outlook.com (A.B.L.); 3 Department of Biostatistics and Medical Informatics, Faculty of Medicine, Inonu University, 44280 Malatya, Türkiye; seyma.yasar@inonu.edu.tr; 4 Department of Pharmacology, Faculty of Medicine, Inonu University, 44280 Malatya, Türkiye; onural.ozhan@inonu.edu.tr (O.O.); mertugrul.balkar@inonu.edu.tr (M.E.B.); hakan.parlakpinar@inonu.edu.tr (H.P.); 5 Department of Cardiology, Faculty of Medicine, Inonu University, 44280 Malatya, Türkiye; zeynep.ulutas@inonu.edu.tr; 6 Department of Physiology, Faculty of Medicine, Malatya Turgut Ozal University, 44210 Malatya, Türkiye; dogrufeyzi@hotmail.com

**Keywords:** acute kidney injury, cecal ligation and puncture, molsidomine, nitric oxide, oxidative stress, sepsis

## Abstract

Sepsis-associated renal injury involves oxidative, inflammatory, and microvascular disturbances. This study evaluated renal changes associated with molsidomine pretreatment in cecal ligation and puncture (CLP)-induced polymicrobial sepsis. Forty Wistar rats were randomly allocated to Sham (n = 8), molsidomine (MOL; n = 8), CLP (n = 12), or MOL+CLP (n = 12) groups. Molsidomine (10 mg/kg/day) was administered orally for 14 days before surgery. Four animals in each septic group died before 24 h, leaving eight survivors per group. The primary outcome was total renal histopathological damage score; secondary outcomes included renal biochemical and immunohistochemical markers and serum renal indices. The total histopathological score differed across groups (overall *p* < 0.001) and was lower in surviving MOL+CLP than CLP animals (2.22 ± 0.97 vs. 5.38 ± 1.06). All four injury components were numerically lower, but adjusted pairwise differences were significant only for glomerular capillary collapse and renal tubular injury. Compared with CLP, MOL+CLP showed lower renal malondialdehyde (106.41 ± 9.03 vs. 136.45 ± 20.27 nmol/g), higher superoxide dismutase and glutathione peroxidase activities, and lower VEGF and P-selectin immunoreactivity. Renal nitric oxide, other redox and inflammatory markers, and serum creatinine, blood urea nitrogen, and urea did not differ significantly between septic groups after multiplicity adjustment. Among survivors, molsidomine pretreatment was associated with less pronounced renal structural injury and tissue-level abnormalities, without significant differences in conventional serum renal indices. Its effects when initiated after sepsis onset remain unknown. Further studies should evaluate post-sepsis dosing, antimicrobial and supportive care, and longer-term renal functional outcomes.

## 1. Introduction

Sepsis is a life-threatening syndrome caused by a dysregulated host response to infection that results in acute organ dysfunction [1]. Despite advances in antimicrobial and supportive care, sepsis remains a major cause of morbidity and mortality worldwide [2]. Sepsis-induced organ injury arises from interconnected inflammatory, metabolic, and vascular disturbances. In particular, oxidative and nitrosative stress, endothelial activation, and microcirculatory dysfunction disrupt cellular homeostasis and tissue oxygen utilization [3]. The kidney is particularly vulnerable to these insults, making sepsis-associated renal injury an important manifestation of acute organ dysfunction [4].

Sepsis-associated acute kidney injury (SA-AKI) is a common and heterogeneous complication of sepsis associated with adverse short- and long-term outcomes [4]. Current concepts extend beyond global renal hypoperfusion, emphasizing dysregulated inflammation, microcirculatory alterations, metabolic reprogramming, and tubular stress [5]. Endothelial activation can increase vascular permeability, leukocyte–endothelial interactions, and microvascular flow heterogeneity, impairing tissue oxygen and nutrient delivery to the kidney. Concurrently, excessive reactive oxygen species generation and impaired antioxidant defenses can disrupt mitochondrial function and tubular epithelial homeostasis [6]. Together, these vascular, inflammatory, and redox disturbances contribute to septic renal injury and provide a rationale for investigating renal redox alterations and endothelial activation markers in experimental sepsis.

Experimental models are essential for investigating sepsis pathobiology and pharmacological responses under controlled conditions [7]. Cecal ligation and puncture (CLP) is an established experimental model of polymicrobial sepsis that generates a sustained intra-abdominal infectious focus and reproduces key systemic features of human sepsis [7]. CLP also produces renal functional and structural alterations and is widely used to investigate sepsis-associated renal injury [8]. Given the contribution of vascular and redox disturbances to septic renal injury, nitric oxide signaling represents a relevant pathway for investigation in this model [9].

Nitric oxide (NO) has a context-dependent role in septic renal pathophysiology, determined by its cellular source, concentration, and redox environment [9]. Under physiological conditions, constitutive NO production through endothelial and neuronal nitric oxide synthases regulates renal vascular tone, intrarenal blood flow, and glomerular hemodynamics [9]. During sepsis, upregulation of inducible nitric oxide synthase (iNOS) may increase NO production and alter its spatial distribution within the kidney [10]. In an oxidant-rich environment, NO reacts with superoxide to generate peroxynitrite, which can contribute to protein modification, mitochondrial dysfunction, and tubular injury [11]. Thus, the renal effects of NO in sepsis depend on the interplay between physiological NO signaling and oxidant-driven nitrosative injury, rather than on total NO abundance alone.

Molsidomine is a sydnonimine-class vasodilator and nitric oxide donor used clinically for the prophylactic treatment of angina pectoris [12]. It is converted to the active metabolite linsidomine (SIN-1), which spontaneously decomposes to release NO and thereby stimulates the soluble guanylate cyclase–cyclic guanosine monophosphate (cGMP) signaling pathway [12]. Unlike organic nitrates, NO release from sydnonimines does not require thiol-dependent enzymatic bioactivation and is less susceptible to pharmacological tolerance [13]. Experimental studies also indicate that molsidomine can modulate renal oxidative stress, antioxidant defenses, and structural injury in experimental models of renal damage [14]. These properties provide a rationale for investigating whether prophylactic molsidomine pretreatment modifies renal redox responses and tissue injury in CLP-induced polymicrobial sepsis, an experimental setting in which its effects remain insufficiently characterized.

Accordingly, the present study aimed to investigate the effects of molsidomine pretreatment on renal injury and associated biochemical and immunohistochemical responses to cecal ligation and puncture-induced polymicrobial sepsis. We evaluated renal redox and inflammatory markers, histopathological injury, and VEGF and P-selectin immunoreactivity to characterize the biochemical and tissue-level changes associated with pretreatment. The total renal histopathological damage score was designated as the primary outcome, while renal biochemical, immunohistochemical, and serum-based renal functional indices were assessed as secondary outcomes.

## 2. Results

### 2.1. Experimental Animal Flow and Mortality

A total of 40 rats were allocated to four experimental groups: Sham (n = 8), MOL (n = 8), CLP (n = 12), and MOL+CLP (n = 12). All animals in the Sham and MOL groups survived to the 24 h endpoint. Four animals in each septic group died before the scheduled endpoint, corresponding to a mortality rate of 33.3% in both CLP and MOL+CLP groups. Consequently, 32 animals completed the experimental protocol, with eight surviving animals in each group available for renal tissue assessment.

### 2.2. Renal Histopathological Injury: Primary Outcome

The primary outcome, total renal histopathological damage score, differed significantly across the four experimental groups (overall *p* < 0.001). Among animals surviving to the 24 h endpoint, the score was highest in the CLP group and was lower in the MOL+CLP group (mean ± SD, 5.38 ± 1.06 vs. 2.22 ± 0.97, respectively) (Table 1). The difference between the two septic groups was also significant in the adjusted pairwise comparison reported in Table 1. Sham kidneys showed preserved renal architecture, whereas CLP was associated with tubular injury, interstitial inflammatory cell infiltration, and vascular congestion. These histopathological alterations were less pronounced in the MOL+CLP group.

**Figure 1:**
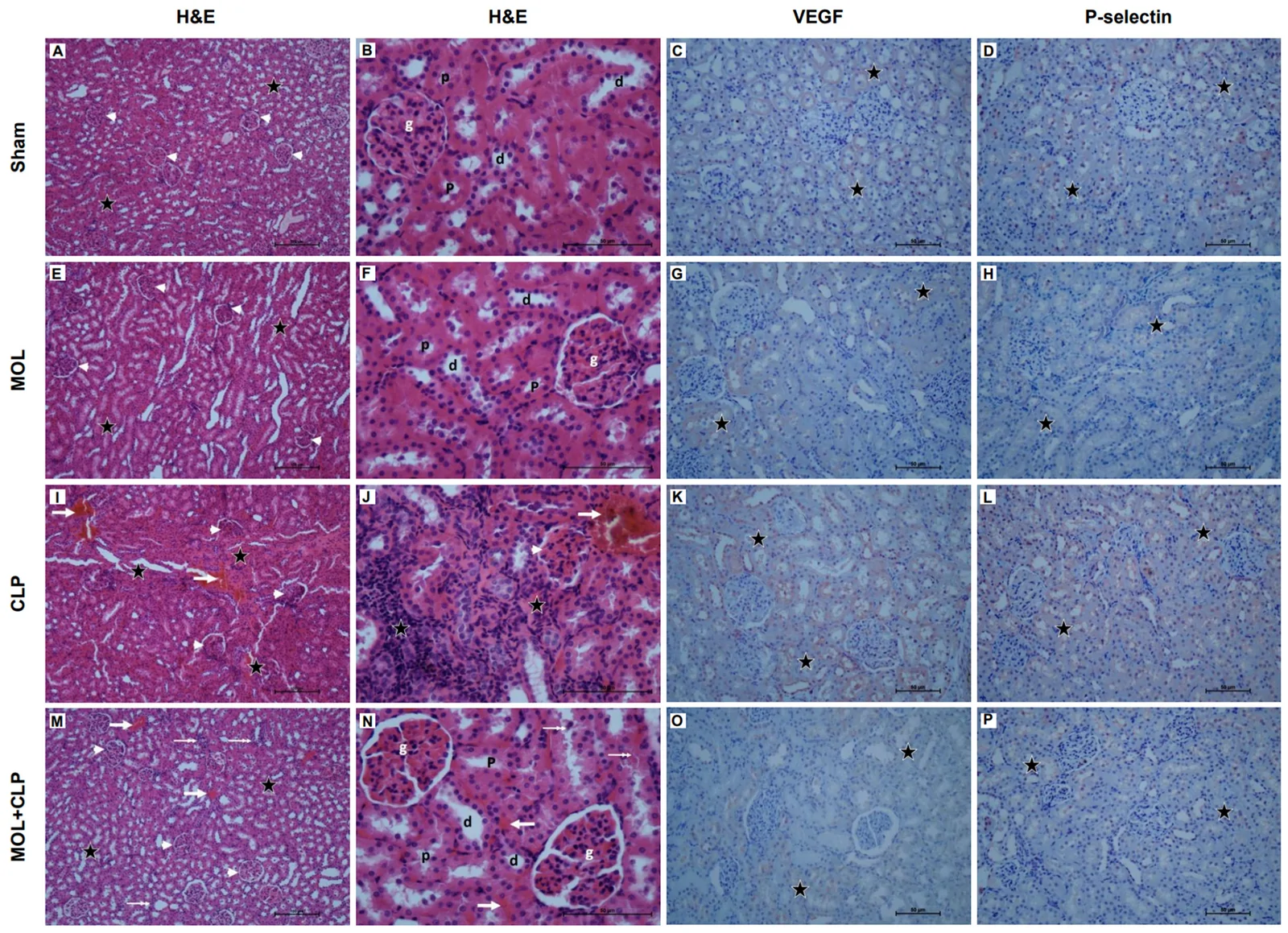
Representative renal histopathological and immunohistochemical findings across the experimental groups. (**A**–**D**) Sham group; (**E**–**H**) MOL group; (**I**–**L**) CLP group; and (**M**–**P**) MOL+CLP group. The first and second columns show representative H&E-stained renal sections at lower and higher magnification, respectively; the third and fourth columns show VEGF and P-selectin immunohistochemical staining, respectively. Sham and MOL kidneys show preserved glomerular and tubular architecture (**A**,**B**,**E**,**F**). CLP kidneys show prominent tubular injury and necrosis, interstitial inflammatory cell infiltration, and vascular congestion (**I**,**J**), whereas these changes are less pronounced in MOL+CLP kidneys (**M**,**N**). VEGF and P-selectin immunoreactivity is increased in the CLP group (**K**,**L**) and lower in the MOL+CLP group (**O**,**P**). In H&E sections, g, p, and d denote glomerulus, proximal tubule, and distal tubule, respectively. Arrowheads identify glomeruli/glomerular alterations, single arrows indicate vascular congestion, and double-headed arrows indicate areas of tubular epithelial injury or inflammatory cell infiltration where shown. Asterisks identify representative renal tubular or immunoreactive areas according to the corresponding panel. Scale bars = 100 μm in (**A**,**E**,**I**,**M**) and 50 μm in (**B**–**D**,**F**–**H**,**J**–**L**,**N**–**P**). CLP, cecal ligation and puncture; H&E, hematoxylin and eosin; MOL, molsidomine; VEGF, vascular endothelial growth factor.

Glomerular capillary collapse, renal tubular injury, interstitial inflammation, and vascular congestion each differed significantly across the experimental groups (overall *p* < 0.001 for all four components), with higher mean scores in the CLP group than in the MOL+CLP group (Table 1). For CLP versus MOL+CLP, the respective mean ± SD scores were 1.00 ± 0.00 versus 0.22 ± 0.44 for glomerular capillary collapse, 1.38 ± 0.52 versus 0.56 ± 0.53 for renal tubular injury, 1.63 ± 0.52 versus 0.78 ± 0.67 for interstitial inflammation, and 1.38 ± 0.52 versus 0.67 ± 0.50 for vascular congestion. The adjusted pairwise comparisons indicated significant differences between CLP and MOL+CLP for glomerular capillary collapse and renal tubular injury, but not for interstitial inflammation or vascular congestion. Representative renal histopathological findings across the four groups are shown in Figure 1.

### 2.3. Renal Oxidative Stress, Antioxidant Status, and Inflammatory Markers

Renal MDA concentrations differed significantly among the experimental groups (overall *p* < 0.001). MDA was higher in the CLP group than in the Sham and MOL groups and lower in the MOL+CLP group than in the CLP group (136.45 ± 20.27 vs. 106.41 ± 9.03 nmol/g) (Table 2). NO and GSH also differed significantly among groups (overall *p* = 0.004 and *p* = 0.030, respectively), but neither differed significantly between the CLP and MOL+CLP groups after multiplicity-adjusted pairwise comparisons. For CLP versus MOL+CLP, NO concentrations were 26.42 ± 5.88 versus 20.84 ± 4.67 μmol/g, respectively, whereas GSH concentrations were 37.03 (6.28) versus 40.36 (1.63) μmol/g [median (IQR)].

SOD and GPx activities differed significantly among groups (overall *p* < 0.001 for both) and were higher in the MOL+CLP group than in the CLP group (SOD: 36.23 ± 4.19 vs. 24.12 ± 2.23 U/g protein; GPx: 92.96 (18.68) vs. 59.32 (12.20) U/mg protein [median (IQR)]). CAT and TAS did not differ significantly among groups (*p* = 0.157 and *p* = 0.364, respectively). TOS, OSI, and TNF-α showed significant overall group differences (*p* = 0.009, *p* = 0.004, and *p* = 0.045, respectively), but none differed significantly between the CLP and MOL+CLP groups after multiplicity-adjusted pairwise comparisons (Table 2). Thus, the differences between the two septic groups were confined to MDA, SOD, and GPx among the renal biochemical markers assessed; the remaining redox and inflammatory indices did not show a statistically significant response to molsidomine pretreatment in this comparison.

### 2.4. Serum Renal Biochemical Findings

Serum BUN and urea concentrations differed significantly among the experimental groups (overall *p* = 0.025 for both) and were higher in the CLP and MOL+CLP groups than in the Sham and MOL groups. However, the adjusted pairwise comparisons showed that CLP differed significantly from Sham and MOL, whereas MOL+CLP differed significantly from MOL but not from Sham. For CLP versus MOL+CLP, mean ± SD values were 39.07 ± 27.41 versus 36.33 ± 21.53 mg/dL for BUN and 83.60 ± 58.65 versus 77.75 ± 46.08 mg/dL for urea, respectively. Serum creatinine also differed significantly among groups (overall *p* = 0.004), with a higher concentration in the CLP group than in the Sham group (mean ± SD, 0.59 ± 0.23 vs. 0.36 ± 0.05 mg/dL). Creatinine was also significantly higher in the CLP group than in the MOL group, whereas the MOL+CLP group did not differ significantly from either CLP or MOL. However, no significant differences were observed between the CLP and MOL+CLP groups for serum creatinine, BUN, or urea after multiplicity-adjusted pairwise comparisons (Supplementary Table S1). Thus, the attenuation of renal histopathological injury and selected tissue redox abnormalities in the MOL+CLP group was not accompanied by a statistically significant improvement in the conventional serum renal biochemical indices measured at 24 h.

### 2.5. Renal Immunohistochemical Findings

Renal VEGF and P-selectin immunoreactivity differed significantly among the experimental groups (overall *p* < 0.001 for both). VEGF H-scores were lower in the MOL+CLP group than in the CLP group (47.5 ± 10.35 vs. 135.0 ± 20.70), with no significant difference between the MOL+CLP and Sham groups. VEGF H-scores were also significantly higher in the MOL+CLP group than in the MOL group (47.5 ± 10.35 vs. 27.5 ± 7.07). Similarly, P-selectin H-scores were lower in the MOL+CLP group than in the CLP group (37.5 ± 10.35 vs. 135.0 ± 14.14), whereas the Sham, MOL, and MOL+CLP groups did not differ significantly (Table 3). These findings indicate lower renal VEGF and P-selectin immunoreactivity in the MOL+CLP group than in the CLP group among animals surviving to the 24 h endpoint, although VEGF immunoreactivity remained significantly higher than in the MOL-only group. Representative renal VEGF and P-selectin immunostaining across the experimental groups is shown in Figure 1.

## 3. Discussion

The principal finding of this study was that molsidomine pretreatment was associated with less pronounced renal structural injury in CLP-induced polymicrobial sepsis among animals surviving to the 24 h endpoint. The prespecified total renal histopathological damage score was lower in the MOL+CLP group, with numerically lower scores across all four histopathological components, although adjusted pairwise differences were significant only for glomerular capillary collapse and renal tubular injury. Molsidomine pretreatment was also associated with lower renal MDA concentrations and higher SOD and GPx activities, although no significant differences between the septic groups were observed for several other redox and inflammatory indices. VEGF and P-selectin immunoreactivity were likewise markedly attenuated, but these immunohistochemical findings do not establish improved endothelial function. Overall, molsidomine pretreatment was associated with attenuation of renal structural injury and selected redox and immunohistochemical abnormalities, but these findings were not accompanied by statistically significant differences between the septic groups in conventional serum renal biochemical indices.

The marked reduction in total renal histopathological damage score provides the strongest evidence of attenuated renal structural injury associated with prophylactic molsidomine administration among animals surviving to the 24 h endpoint. Contemporary concepts describe SA-AKI as a heterogeneous process in which tubular, inflammatory, metabolic, and microvascular disturbances interact rather than reflecting renal hypoperfusion alone [15]. Oxidative stress can contribute to renal cellular injury through mitochondrial dysfunction, impaired energy metabolism, and disruption of cellular homeostasis [16]. Endothelial activation further contributes to renal injury by promoting vascular permeability, leukocyte–endothelial interactions, and microcirculatory dysfunction [17]. Against this mechanistic background, the lower total damage score, together with significantly lower glomerular capillary collapse and renal tubular injury scores and numerically lower interstitial inflammation and vascular congestion scores, is consistent with attenuation of renal structural injury associated with molsidomine pretreatment in this experimental model. However, these histopathological findings do not establish improved renal perfusion or functional recovery.

CLP increased renal MDA and reduced SOD and GPx activities, whereas molsidomine pretreatment lowered MDA and increased both antioxidant enzyme activities, suggesting attenuation of lipid peroxidation and preservation of selected renal antioxidant defenses rather than restoration of overall redox homeostasis. Similarly, Xia et al. reported that TAK-242 reduced renal MDA and reactive oxygen species, increased SOD activity, and ameliorated renal injury in experimental SA-AKI through inhibition of TLR4/NF-κB signaling [18]. Zhu et al. demonstrated that sivelestat reduced MDA while increasing SOD and GPx activities and attenuating renal histopathological injury after CLP [19]. Karaboğa et al. likewise showed that ebselen ameliorated sepsis-induced renal injury through modulation of oxidative stress together with endoplasmic reticulum stress and apoptosis in an LPS-induced rat model [20]. Collectively, these studies support oxidative imbalance as an important component of septic renal injury and provide a consistent experimental context for the selective redox changes observed with molsidomine. However, the absence of significant differences between CLP and MOL+CLP in renal GSH, CAT, TAS, TOS, OSI, and TNF-α indicates that molsidomine did not produce a uniform biochemical response across the measured pathways.

Molsidomine pretreatment was not associated with statistically significant differences in renal NO, GSH, TOS, OSI, or TNF-α, or in serum creatinine, BUN, or urea compared with untreated septic animals, indicating a non-uniform renal response. This distinction is particularly important because the primary finding of our study was a lower renal histopathological damage score, whereas statistically significant differences in conventional serum renal biochemical indices between the septic groups were not demonstrated at the 24 h endpoint. Wiersema et al. identified two clinical septic AKI subphenotypes with distinct inflammatory and endothelial injury profiles, renal recovery, and 90-day mortality [21]. Molinari et al. demonstrated that urinary tubular stress biomarkers can provide prognostic information beyond conventional functional AKI staging in patients with septic shock [22]. Similarly, the PHENAKI study identified three distinct clinical SA-AKI subphenotypes by integrating kidney function variables with urinary [TIMP-2] × [IGFBP7] measurements [23]. Thus, the coexistence of reduced renal histopathological injury and selected redox abnormalities with no statistically significant differences in conventional serum renal biochemical indices between the septic groups is biologically plausible; however, these clinical studies do not establish that molsidomine improved renal function or altered an AKI subphenotype in our experimental model. Additional measurements of glomerular filtration, urine output, and tubular injury or stress biomarkers would be required to determine whether the observed tissue-level differences translate into functional renal benefit.

The renal structural and selective redox changes associated with molsidomine pretreatment in our CLP model have parallels in experimental kidney injury models with different initiating insults. Chander and Chopra showed that molsidomine attenuated renal dysfunction, morphological injury, and oxidative stress in rhabdomyolysis-induced myoglobinuric acute renal failure [24]. Rodriguez-Peña et al. reported reduced renal dysfunction, inflammatory cell infiltration, and adhesion-molecule expression, including P-selectin, following intrarenal molsidomine administration in renal ischemia–reperfusion injury [25]. Karakoc et al. similarly demonstrated reduced tubular degeneration and inflammatory histological changes in cisplatin nephrotoxicity, although the changes in MDA, SOD, CAT, and GPx did not reach statistical significance; unlike our findings, serum BUN and creatinine were also reduced in that model [26]. Together, these studies support the biological plausibility of molsidomine-associated attenuation of renal tissue injury while illustrating that biochemical and functional responses vary across experimental models. Our findings extend this experimental literature to renal structural injury following CLP-induced polymicrobial sepsis, but do not establish functional renal recovery or therapeutic efficacy after sepsis onset.

Our histopathological and immunohistochemical findings further support the involvement of endothelial and microvascular alterations in septic renal injury. Molema et al. characterized renal microvascular endothelial dysfunction in SA-AKI by altered perfusion, permeability, endothelial signaling, and inflammatory interactions [17]. Gupta et al. reported that molsidomine attenuated renal lipid peroxidation and morphological injury in an iron-induced nephrotoxicity model, accompanied by improvements in serum creatinine and BUN [27]. In our study, molsidomine pretreatment was associated with significantly lower renal VEGF and P-selectin immunoreactivity and a lower total histopathological damage score than untreated CLP. Vascular congestion scores were also numerically lower, although the adjusted pairwise difference was not statistically significant. These findings are consistent with attenuation of selected tissue-level abnormalities but do not establish improved renal microvascular perfusion or endothelial function.

The effects of molsidomine should also be interpreted within the context-dependent biology of nitric oxide. Lundberg and Weitzberg emphasized that NO signaling depends on its site of production, local bioavailability, and biochemical environment, with physiological and potentially injurious consequences under different conditions [28]. In the kidney, NO signaling contributes to the regulation of vascular tone and intrarenal perfusion, whereas sepsis-associated microcirculatory and metabolic disturbances may alter renal oxygen delivery and utilization [29]. Betrie et al. showed that renal arterial tempol administration prevented medullary hypoperfusion, hypoxia, and AKI during ovine Gram-negative sepsis and attenuated renal medullary eNOS Thr-495 phosphorylation; notably, these effects occurred without improvement in the measured oxidative/nitrosative stress markers [30]. In our study, renal NO did not differ significantly between CLP and MOL+CLP, despite lower renal MDA, higher SOD and GPx activities, and reduced VEGF and P-selectin immunoreactivity. These findings are compatible with a context-dependent renal response to molsidomine pretreatment but do not demonstrate altered NO bioavailability or downstream NO signaling. Because eNOS/iNOS activity, soluble guanylate cyclase–cGMP signaling, peroxynitrite formation, and renal microvascular perfusion were not directly assessed, the mechanistic relationship between molsidomine, NO signaling, and the observed renal structural changes remains unresolved.

The translational interpretation of our findings requires caution because molsidomine was administered for 14 days before CLP rather than after sepsis onset. Osuchowski et al. emphasized that clinically relevant treatment timing, supportive care, and organ dysfunction endpoints are essential for improving the translational value of preclinical sepsis models [31]. The present pretreatment design was intended to examine whether prior exposure to molsidomine modified the early renal response to a subsequent septic insult; it was not designed to evaluate treatment initiated after sepsis had developed. Moreover, no antibiotics were administered, and renal outcomes were assessed only at 24 h among surviving animals, limiting the applicability of these findings to patients receiving contemporary sepsis care. Guillon et al. highlighted important differences between rodent models and ICU patients, including comorbidities and supportive care [32]. Marshall underscored the recurrent failure of experimental sepsis therapies to translate successfully into clinical practice [33]. Accordingly, our findings provide preclinical evidence of attenuated renal structural injury and selected redox and immunohistochemical abnormalities after prophylactic molsidomine administration, but not evidence of improved renal function or therapeutic efficacy after sepsis onset. Future studies should evaluate post-CLP treatment, clinically relevant antimicrobial and supportive care, renal functional endpoints, and longer-term outcomes, while accounting for deaths before tissue collection.

### Limitations

This study has several limitations. The CLP model cannot fully reproduce the complexity of human sepsis, particularly because antibiotics and comprehensive supportive care were not provided. The 14-day molsidomine pretreatment design does not establish therapeutic efficacy after sepsis onset. Only male animals, a single dose, and a 24 h endpoint were evaluated. Four of 12 animals died in each septic group before tissue collection; consequently, analyses were restricted to survivors and may be affected by survivor bias.

Direct measures of glomerular filtration, urine output, and renal hemodynamics were not available. The reduction in histopathological injury was not accompanied by significant improvement in serum creatinine, BUN, or urea, and longer-term renal recovery could not be assessed. Formal histopathological scoring reproducibility was not established. The molecular mechanisms underlying the observed changes, including eNOS/iNOS activity, sGC–cGMP signaling, and nitrosative stress, were not directly investigated. Reduced VEGF and P-selectin immunoreactivity therefore cannot be taken as direct evidence of improved endothelial function.

## 4. Materials and Methods

### 4.1. Study Design, Ethics, and Reporting

This randomized, controlled experimental study evaluated the effects of prophylactic molsidomine pretreatment on renal injury in a rat model of cecal ligation and puncture (CLP)-induced polymicrobial sepsis. The protocol was approved by the Inonu University Local Ethics Committee for Animal Experiments (Approval No. 2024/17-2; approval date: 18 October 2024, Malatya, Türkiye). All procedures were conducted in accordance with the approved experimental protocol and institutional animal-care guidelines, and the manuscript was prepared with reference to the ARRIVE 2.0 guidelines [34].

### 4.2. Animal Attrition and Analysis Populations

Forty animals were randomly assigned to the Sham, MOL, CLP, and MOL+CLP groups in the ratios of 8, 8, 12, and 12, respectively. Although 12 rats were initially assigned to each of the two CLP groups (CLP and MOL+CLP), four animals in each CLP group died postoperatively before the planned 24 h endpoint was reached. Therefore, the analyses of outcomes assessed at the 24 h endpoint were performed using the eight surviving animals in each group (n = 32 in total).

### 4.3. Animals, Randomization, and Experimental Groups

Forty male Wistar albino rats (10–12 weeks; 256–348 g) were obtained from the Inonu University Experimental Animal Research and Production Center and housed under controlled conditions (25 ± 2 °C, 60 ± 5% relative humidity, 12 h light/dark cycle) with ad libitum access to standard chow and water. The animals were randomly allocated to four experimental groups: Sham (n = 8), MOL (n = 8), CLP (n = 12), and MOL+CLP (n = 12). The animals were socially grouped, with four rats per cage. The two septic groups were assigned additional animals because mortality following CLP was anticipated. These group sizes represent the initial allocation, whereas the numbers of animals contributing to each endpoint are reported separately under “Animal Attrition and Analysis Populations.” No predefined inclusion or exclusion criteria were applied to the animals used in this study.

### 4.4. Molsidomine Pretreatment and Cecal Ligation and Puncture Procedure

Molsidomine (10 mg/kg/day; Sigma-Aldrich, St. Louis, MO, USA; CAS No.: 25717-80-0) was administered by oral gavage once daily for 14 days before surgery; the oral dose was informed by previous experimental evidence in rat models of renal injury [35], whereas the 14-day pretreatment schedule was specific to the present experimental protocol. MOL and MOL+CLP rats received molsidomine, whereas Sham and CLP rats received saline during the corresponding pretreatment period. For CLP, rats were anesthetized with intraperitoneal ketamine (100 mg/kg) and xylazine (10 mg/kg), prior to surgery, the abdominal region was disinfected with 10% povidone–iodine solution (Batticon antiseptic, ADEKA Pharmaceuticals, Istanbul, Türkiye), followed by a 1 cm midline laparotomy. The cecum was exteriorized, ligated distal to the ileocecal junction without intestinal obstruction, punctured twice with a sterile 22-gauge needle, and gently compressed to extrude a small amount of fecal material before abdominal closure. Sham and MOL rats underwent laparotomy and cecal manipulation without ligation or puncture. CLP and MOL+CLP rats received 1 mL sterile saline subcutaneously for fluid resuscitation. No antibiotics or analgesic were administered to the animals.

### 4.5. Physiological Assessment, Blood Sampling, and Serum Biochemistry

At 24 h after CLP or sham surgery, surviving animals were anesthetized with intraperitoneal ethyl carbamate (1.2 g/kg) (CAS No.: 51-79-6, Urethane^®^, Sigma-Aldrich, St. Louis, MO, USA). Blood was collected from the inferior vena cava and centrifuged at 2500 rpm for 7 min. Serum creatinine, blood urea nitrogen (BUN), and urea were measured as indices of renal function. Animals were then euthanized by exsanguination, and the kidneys were collected for biochemical, histopathological, and immunohistochemical analyses. Kidney samples intended for biochemical analyses were stored at −80 °C, whereas specimens intended for histopathological and immunohistochemical evaluation were fixed in 10% neutral buffered formalin.

### 4.6. Renal Tissue Biochemical Analyses

Kidney tissues were homogenized and centrifuged to obtain supernatants for biochemical analyses. Malondialdehyde (MDA) was measured spectrophotometrically using the Uchiyama–Mihara method [36] and expressed as nmol/g tissue. Nitric oxide (NO) was assessed from stable nitrite/nitrate metabolites using the Griess reaction [37] and expressed as µmol/g tissue. Reduced glutathione (GSH) was determined using Ellman’s method [38] and expressed as µmol/g tissue. Tissue protein concentration was determined using the modified Lowry method [39] for normalization of antioxidant enzyme activities. Superoxide dismutase (SOD) activity was determined spectrophotometrically using the method of Sun and colleagues [40] and expressed as U/g protein. Catalase (CAT) activity was measured using Aebi’s method [41] and expressed as K/g protein. Glutathione peroxidase (GPx) activity was determined using the Paglia–Valentine method [42] and expressed as U/mg protein. Total oxidant status (TOS) was determined using Erel’s automated colorimetric method [43] and expressed as µmol H_2_O_2_ equivalent/L, and total antioxidant status (TAS) was measured using Erel’s automated colorimetric method [44] and expressed as mmol Trolox equivalent/L. The oxidative stress index (OSI) was calculated as the TOS/TAS ratio after accounting for the different concentration units and expressed in arbitrary units. Renal tissue tumor necrosis factor-alpha (TNF-α) was measured using a rat-specific ELISA kit (YL Biont, Shanghai YL Biotech Co., Ltd., Shanghai, China; catalog no. YLA0118RA) according to the manufacturer’s instructions.

### 4.7. Histopathological Examination

Kidney specimens were fixed in 10% neutral buffered formalin for 48 h, paraffin-embedded, sectioned at 5–6 µm, and stained with hematoxylin and eosin (H&E). Four H&E sections from each animal were examined by an experienced histologist blinded to group allocation; slide labels were covered before microscopic evaluation. The entire section areas were examined using ×4, ×10, and ×40 objectives. Four renal injury components were assessed: glomerular capillary collapse, renal tubular injury, interstitial inflammation, and vascular congestion. Glomerular capillary collapse was evaluated in 100 randomly selected glomeruli across the four sections. Renal tubular injury was assessed according to the proportion of injured tubules within the examined section area, whereas interstitial inflammation and vascular congestion were evaluated semi-quantitatively according to their frequency and extent. Each component was scored on a 0–3 scale, with higher scores indicating more severe injury. For each animal, component scores obtained from the examined sections were averaged, and the four resulting component scores were summed to obtain the total renal histopathological damage score, ranging from 0 to 12. Histopathological examinations were scored by a single blinded observer.

### 4.8. Immunohistochemical Analysis

Immunohistochemistry was performed on 4 µm kidney sections to evaluate vascular endothelial growth factor (VEGF) and P-selectin expression. Following antigen retrieval in citrate buffer (pH 6.0), sections were incubated overnight at 4 °C with primary antibodies against VEGF (Santa Cruz Biotechnology, Dallas, TX, USA; C-1, sc-7269; 1:100) and P-selectin (Santa Cruz Biotechnology, USA; sc-8419; 1:200). Immunoreactivity was visualized using biotinylated secondary antibodies and 3,3′-diaminobenzidine (DAB). Immunostaining was evaluated by an observer blinded to group allocation using the semi-quantitative H-score: Σ[Pi × (i + 1)], where Pi represents the percentage of cells at each staining intensity and i represents the corresponding intensity score (0–3).

### 4.9. Study Outcomes and Sample-Size Considerations

The individual animal was considered the experimental unit. The total renal histopathological damage score was defined as the primary outcome, and comparisons among all groups constituted the main focus of the study. Secondary outcomes included renal redox and inflammatory indices, individual histopathological components, VEGF and P-selectin immunoreactivity, serum renal biochemical indices, and physiological variables. Sample size was calculated a priori (G*Power v3.1.9.7) using a one-way ANOVA (F test) for four groups. Assuming a large effect size (f = 0.65), α = 0.05, and 80% power, a minimum of 8 animals per group (32 total) was required (achieved power = 0.831). Anticipating up to 50% CLP-related mortality, septic groups were set at n = 12, totaling 40.

### 4.10. Statistical Analysis

To ensure objectivity during data analysis and eliminate potential biases, the researcher who performed the statistical analyses was blinded to the group assignments. Descriptive statistics are presented as the mean ± standard deviation and median (IQR) for numerical data and as a count (percentage) for categorical data. The normality of continuous variables was assessed using the Shapiro–Wilk test. Intergroup comparisons were performed using one-way analysis of variance (ANOVA) or the Kruskal–Wallis test, depending on the distribution characteristics. For variables with statistically significant differences in general tests, post hoc pairwise comparisons were performed; for parametric data, variance homogeneity after ANOVA was assessed using the Levene test, Tukey HSD was used when variances were homogeneous, the Tamhane T2 post hoc test was used when they were not homogeneous, and the Mann–Whitney U test with Bonferroni correction was used for non-parametric data. *p* < 0.05 was considered statistically significant. All statistical analyses were performed using IBM SPSS Statistics for Windows, Version 26.0 (IBM Corp., Armonk, NY, USA).

## 5. Conclusions

Molsidomine pretreatment was associated with less pronounced renal structural injury, changes in selected oxidative stress markers, and lower renal VEGF and P-selectin immunoreactivity in animals surviving to the 24 h endpoint after CLP-induced polymicrobial sepsis. These findings suggest that pretreatment may modify selected renal tissue-level responses to sepsis. However, several other biochemical markers and conventional serum renal indices did not differ significantly between the septic groups, and the observed tissue-level changes cannot be assumed to indicate early functional improvement. Given the prophylactic design, these preliminary findings do not establish therapeutic effects when molsidomine is initiated after sepsis onset. Further studies incorporating post-sepsis treatment, clinically relevant antimicrobial and supportive care, and longer-term renal structural and functional assessments may help clarify the potential role of molsidomine in sepsis-associated renal injury.

## Figures and Tables

**Table 1:** Renal histopathological injury scores across experimental groups.

	Group								*p*-Value ^0^
	SHAM		MOL		CLP		MOL+CLP		
	Mean ± SD	Median (IQR)	Mean ± SD	Median (IQR)	Mean ± SD	Median (IQR)	Mean ± SD	Median (IQR)	
Glomerular Capillary Collapse	0 ^a^ ± 0	0 (0)	0 ^a^ ± 0	0 (0)	1 ^b^ ± 0	1 (0)	0.22 ^a^ ± 0.44	0 (0)	<0.001 *
Renal Tubular Injury	0 ^a^ ± 0	0 (0)	0 ^a^ ± 0	0 (0)	1.38 ^b^ ± 0.52	1 (1)	0.56 ^c^ ± 0.53	1 (1)	<0.001 *
Interstitial Inflammation	0 ^a^ ± 0	0 (0)	0 ^a^ ± 0	0 (0)	1.63 ^b^ ± 0.52	2 (1)	0.78 ^b^ ± 0.67	1 (1)	<0.001 *
Vascular Congestion	0 ^a^ ± 0	0 (0)	0 ^a^ ± 0	0 (0)	1.38 ^b^ ± 0.52	1 (1)	0.67 ^b^ ± 0.5	1 (1)	<0.001 *
Total Damage Score	0 ^a^ ± 0	0 (0)	0 ^a^ ± 0	0 (0)	5.38 ^b^ ± 1.06	5 (0.5)	2.22 ^c^ ± 0.97	2 (1)	<0.001 *

^0^: There is a statistically significant difference in group categories that do not contain the same letter. *: Kruskal–Wallis H test. Abbreviations: CLP, cecal ligation and puncture; IQR, interquartile range; MOL, molsidomine.

**Table 2:** Renal tissue oxidative stress, antioxidant defense, and inflammatory markers across experimental groups.

	Group								*p*-Value ^0^
	SHAM		MOL		CLP		MOL+CLP		
	Mean ± SD	Median (IQR)	Mean ± SD	Median (IQR)	Mean ± SD	Median (IQR)	Mean ± SD	Median (IQR)	
MDA(nmol/g)	99.13 ^a^ ± 9.96	99.68 (11.57)	107.34 ^a^ ± 11.2	102.96 (10.45)	136.45 ^b^ ± 20.27	140.91 (37.73)	106.41 ^a^ ± 9.03	104.88 (6.96)	<0.001 *
NO (μmol/g)	18.4 ^a^ ± 3.65	18.27 (4.98)	19.6 ^a^ ± 2.42	19.29 (2.45)	26.42 ^b^ ± 5.88	26.44 (8.54)	20.84 ^a,b^ ± 4.67	19.89 (6.09)	0.004 *
GSH (μmol/g)	41.94 ^a^ ± 2.05	41.64 (2.21)	41.2 ^a^ ± 2.35	42.24 (2.28)	36.72 ^b^ ± 4.76	37.03 (6.28)	40.14 ^a,b^ ± 1.68	40.36 (1.63)	0.030 **
SOD (U/g protein)	38.92 ^a^ ± 7.53	35.82 (13.42)	37.49 ^a^ ± 2.84	36.94 (4.1)	24.12 ^b^ ± 2.23	23.41 (3.23)	36.23 ^a^ ± 4.19	35.84 (4.18)	<0.001 *
CAT (K/g protein)	215.1 ^a^ ± 45.39	213.92 (64.61)	221.08 ^a^ ± 68.94	213.44 (98.94)	159.42 ^a^ ± 58.77	137.5 (70.3)	187.52 ^a^ ± 66.48	181.89 (68.51)	0.157 *
GPx (U/mg protein)	96.8 ^a^ ± 11.13	96.56 (11.16)	86.7 ^a^ ± 7.24	87.86 (7.95)	61.54 ^b^ ± 9.1	59.32 (12.2)	99.43 ^a^ ± 26.38	92.96 (18.68)	<0.001 **
TAS (mmol trolox eq/L)	3.35 ^a^ ± 0.34	3.28 (0.4)	3.2 ^a^ ± 0.35	3.21 (0.47)	3.07 ^a^ ± 0.54	2.85 (0.69)	3.03 ^a^ ± 0.24	3.03 (0.38)	0.364 *
TOS (μmol H_2_O_2_ eq/L)	7.29 ^a^ ± 0.73	7.03 (1.15)	8.29 ^a,b^ ± 0.98	7.98 (1.31)	10.56 ^c^ ± 2.31	9.34 (4.18)	9.46 ^b,c^ ± 2.59	9.82 (4.32)	0.009 **
OSİ (AU)	2.2 ^a^ ± 0.37	2.16 (0.6)	2.63 ^a,b^ ± 0.46	2.76 (0.81)	3.51 ^b^ ± 0.88	3.39 (0.79)	3.15 ^a,b^ ± 0.93	3.34 (1.4)	0.004 *
TNF-α (pg/mL)	288.42 ^a^ ± 10.99	288.08 (19.24)	307.88 ^a,b^ ± 45.35	310.46 (37.59)	337.25 ^b^ ± 51.83	325.22 (22.37)	321.75 ^a,b^ ± 54.65	305.09 (73.16)	0.045 **

^0^: Superscript letters (a, b, and c) indicate the results of post hoc pairwise comparisons. Groups sharing at least one letter are not significantly different, whereas groups with no letter in common differ significantly (adjusted *p* < 0.05). *: One-way ANOVA, **: Kruskal–Wallis H test. Abbreviations: AU, arbitrary units; CAT, catalase; CLP, cecal ligation and puncture; GPx, glutathione peroxidase; GSH, reduced glutathione; MDA, malondialdehyde; MOL, molsidomine; NO, nitric oxide; OSI, oxidative stress index; SD, standard deviation; SOD, superoxide dismutase; TAS, total antioxidant status; TNF-α, tumor necrosis factor-alpha; TOS, total oxidant status.

**Table 3:** Renal VEGF and P-selectin immunoreactivity across experimental groups.

	Group								*p*-Value ^0^
	SHAM		MOL		CLP		MOL+CLP		
	Mean ± SD	Median (IQR)	Mean ± SD	Median (IQR)	Mean ± SD	Median (IQR)	Mean ± SD	Median (IQR)	
VEGF Immunoreactivity H Score	32.5 ^a,b^ ± 7.07	30 ^a,b^ (10)	27.5 ^a^ ± 7.07	30 ^a^ (10)	135 ^c^ ± 20.7	140 ^c^ (25)	47.5 ^b^ ± 10.35	50 ^b^ (12.5)	<0.001 *
P Selectin Immunoreactivity H Score	25 ^a^ ± 9.26	25 ^a^ (10)	23.75 ^a^ ± 9.16	20 ^a^ (10)	135 ^b^ ± 14.14	140 ^b^ (20)	37.5 ^a^ ± 10.35	40 ^a^ (12.5)	<0.001 *

^0^: There is a statistically significant difference in group categories that do not contain the same letter. *: One-way ANOVA. Abbreviations: CLP, cecal ligation and puncture; H-score, histochemical score; MOL, molsidomine; SD, standard deviation; VEGF, vascular endothelial growth factor.

## Data Availability

The original contributions presented in this study are included in the article/Supplementary Material. Further inquiries can be directed to the corresponding author.

## Supplementary Materials

The following supporting information can be downloaded at: https://www.mdpi.com/article/10.3390/ijms27209088/s1.
