# Supplementary material for: Molsidomine Pretreatment Modulates Renal Redox and Endothelial Activation Markers in Experimental Polymicrobial Sepsis
Source: Int J Mol Sci. 2026 Oct 14;27(20):9088. doi: 10.3390/ijms27209088 (PMC13705544; doi:10.3390/ijms27209088)
Supplement: Supplementary file 1 [file ijms-27-09088-s001.zip › ijms-4568748-supplementary.pdf]

Supplementary Table S1. Serum renal biochemical indices across experimental groups

	Group								p-value°
	SHAM		MOL		CLP		MOL+CLP		
	Mean±SD	Median(IQR)	Mean±SD	Median(IQR)	Mean±SD	Median(IQR)	Mean±SD	Median(IQR)	
BUN (mg/dL)	20.66a,b±3.32	19.63(3.27)	19.57a±2.82	18.93(3.75)	39.07c±27.41	28.97(16.35)	36.33b,c±21.53	29.21(23.24)	0.025*
Creatinine (mg/dL)	0.36a±0.05	0.4(0.1)	0.38a,b±0.05	0.4(0.03)	0.59c±0.23	0.5(0.07)	0.48b,c±0.16	0.45(0.22)	0.004*
Urea (mg/dL)	44.22a,b±7.1	42(7)	41.88a±6.03	40.5(8)	83.6c±58.65	62(35)	77.75b,c±46.08	62.5(49.75)	0.025*

°: There is a statistically significant difference in group categories that do not contain the same letter.*: Kruskal-Wallis H test.

Abbreviations: BUN, blood urea nitrogen; CLP, cecal ligation and puncture; IQR, interquartile range; MOL, molsidomine.
